# Shared community history strengthens plant diversity effects on below‐ground multitrophic functioning

**DOI:** 10.1111/1365-2656.14241

**Published:** 2025-01-29

**Authors:** Angelos Amyntas, Benoit Gauzens, Marcel Ciobanu, Lara Warnke, Mark Maraun, Jörg‐Alfred Salamon, Mona Merkle, Leonardo Bassi, Justus Hennecke, Markus Lange, Gerd Gleixner, Stefan Scheu, Nico Eisenhauer, Ulrich Brose

**Affiliations:** ^1^ Institute of Biodiversity Friedrich Schiller University Jena Jena Germany; ^2^ German Centre for Integrative Biodiversity Research (iDiv) Halle‐Jena‐Leipzig Leipzig Germany; ^3^ J.F. Blumenbach Institute of Zoology and Anthropology University of Göttingen Göttingen Germany; ^4^ Institute of Biological Research Cluj, National Institute for Research and Development for Biological Sciences Cluj‐Napoca Romania; ^5^ Institute of Ecology and Evolution & Field Station Schapen University of Veterinary Medicine Hannover Hannover Germany; ^6^ Institute of Biology Leipzig University Leipzig Germany; ^7^ Max Planck Institute for Biogeochemistry Jena Germany

**Keywords:** below‐ground, community assembly, community history, detritivory, food webs, herbivory, predation, soil fauna communities

## Abstract

1. The relationship of plant diversity and several ecosystem functions strengthens over time. This suggests that the restructuring of biotic interactions in the process of a community's assembly and the associated changes in function differ between species‐rich and species‐poor communities. An important component of these changes is the feedback between plant and soil community history.
2. In this study, we examined the interactive effects of plant richness and community history on the trophic functions of the soil fauna community. We hypothesized that experimental removal of either soil or plant community history would diminish the positive effects of plant richness on the multitrophic functions of the soil food web, compared to mature communities. We tested this hypothesis in a long‐term grassland biodiversity experiment by comparing plots across three treatments (without plant history, without plant and soil history, controls with ~20 years of plot‐specific community history).
3. We found that the relationship between plant richness and below‐ground multitrophic functionality is indeed stronger in communities with shared plant and soil community history. Our findings indicate that anthropogenic disturbance can impact the functioning of the soil community through the loss of plant species but also by preventing feedbacks that develop in the process of community assembly.

## Graphical abstract

The relationship between plant richness and below‐ground multitrophic functionality was stronger in communities with shared plant and soil community history. This work highlights the obscure role of below‐ground consumers on diversity–ecosystem functioning relationships.

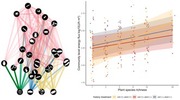

## 1 INTRODUCTION

Changes in biodiversity due to anthropogenic pressure have motivated ecological research to focus on the relationship between biodiversity and ecosystem functioning (BEF) and its relevance for the provision of ecosystem services (Isbell et al., 2017). A plethora of empirical (Cardinale et al., 2011; Hector, 1999; Tilman et al., 1997) and theoretical studies (Albert et al., 2022; Loreau, 1998; Maureaud et al., 2020) has demonstrated that this relationship is generally positive, across different systems (Huang et al., 2018), for several ecosystem functions above‐ as well as below‐ground, indicating that loss of biodiversity would be detrimental to the functioning of ecosystems. There is also mounting evidence that BEF relationships strengthen over time (Huang et al., 2018; Reich et al., 2012), which motivated our study addressing which processes during community assembly could be responsible for this change (Eisenhauer et al., 2019).

The functioning of an ecological community is driven by the biotic interactions of its constituent species (Randall & Smith, 2019), which are in turn influenced by the environment. These interactions change over time, through a combination of plastic adaptations and species turnover processes in response to competition or environmental variability (Agrawal, 2001; Bauer et al., 2022; O'Sullivan et al., 2021). The restructuring of biotic interactions, therefore, shapes the community's history, which influences the level of functioning at different points in time. In that light, diversity can be seen as a crucial context dependency, in the sense that, to understand how functioning will change over time, we need to consider whether the community in question is species‐poor or species‐rich.

Plant species have been shown to shift their traits to facilitate coexistence despite competition (van Moorsel et al., 2018; Zuppinger‐Dingley et al., 2014). This process of niche differentiation among populations in species‐rich plant communities increases complementarity, whereas the potential for this would be reduced in species‐poor communities and not feasible for monocultures. Therefore, plastic or inter‐generational changes of plant niches during the plant community history can be responsible for the steepening of the diversity–productivity relationship (Amyntas et al., 2023). This in turn should enhance soil ecosystem functioning through increased resource input (root biomass, exudates, litter) (Eisenhauer et al., 2013, 2017; Hooper et al., 2000) as well as the concomitant structuring of the soil environment (Frouz, 2024).

However, plant niche partitioning was also shown to depend on soil community composition that may co‐determine eco‐evolutionary processes (van Moorsel et al., 2020; Zuppinger‐Dingley et al., 2015). During assembly, the soil community experiences shifts in species composition, in a turnover process that tends to replace pioneer species (quick colonizers, opportunistic, with a broad niche spectrum) with species that are more competitive and efficient in using resources under stable environmental conditions (Cesarz et al., 2015). Overall, community assembly should lead to a composition of species that are well adapted to the environment and each other. This process also implies a restructuring of trophic interactions in the soil fauna community, which can be highly dependent on the diversity of the underlying plant community (Eisenhauer et al., 2012). High plant diversity offers a variety of niches for the soil fauna, creating the circumstances that would foster a soil community that can maintain higher levels of functioning such as decomposition, herbivory but also control of herbivory by predators (Barnes et al., 2020).

Taken together, the multitrophic functioning of soil fauna should be maximized in plant‐rich communities with plant community history as well as soil history. While there is evidence of a positive effect of plant diversity on trophic functions of above‐ground consumer communities (Barnes et al., 2020; Buzhdygan et al., 2020), this relationship is less clear for below‐ground consumers (Buzhdygan et al., 2020). Moreover, the interactive effects of plant diversity and community history on the functioning of the soil fauna community remain untested so far, in contrast to their influence on the soil microbial community (Schmid et al., 2021). This leads us to the following questions: (a) How does soil community history change the effect of plant diversity on the multitrophic functioning of soil fauna, and (b) how does plant community history change this relationship? We addressed these questions in a large‐scale experiment, manipulating plant coexistence history and soil community history, to examine their effects on the functioning of the soil fauna community. We used energy flux in the soil food web (i.e. the energy required to support an observed multitrophic community) as a proxy of different trophic functions of the soil fauna community (Barnes et al., 2018). This approach allows us to explicitly connect populations of taxa with complex diets to distinct trophic functions such as detritivory or predation (Potapov, 2022) and thereby assess trophic functions that are not possible to measure directly, especially below‐ground. We hypothesized that (H1) plant species richness would increase the overall functioning of the soil fauna across communities with plot‐specific soil and plant history. (H2) This relationship would be incrementally weakened by the removal of plant and soil history. (H3) Consistent with what has been observed above‐ground (Barnes et al., 2020), the restructuring of trophic interactions over time would lead to increased herbivore control at higher plant species richness and reduced herbivory pressure on plants.

## 2 MATERIALS AND METHODS

### 2.1 Experimental field site

The Jena Experiment was established in 2002 in the floodplain of the river Saale (Thuringia, Germany, 50°55′ N, 11°35′ E; 130 m a.s.l.) (Roscher et al., 2004). It is a long‐term biodiversity ecosystem functioning experiment, consisting of 80 grassland plots with maintained plant species richness. Across the plots, sown species richness doubles from 1 to 16 species (each level of richness is replicated 16 times except for 1‐ and 16‐species plots that are replicated 14 times). Additionally, there are four plots sown with all 60 species which comprise the whole species pool of the experiment. Plots are arranged in four blocks. Experimental species richness is maintained by weeding three times per year. Plots are mown twice a year, consistent with typical management practice in Central European extensively used grasslands.

### 2.2 The ΔBEF experiment

In 2016, a split‐plot design was established in each plot of the Jena Experiment (details in Vogel et al., 2019). One subplot was the control, with plot‐specific soil and plant community history and the other two are treatments with a cumulative removal of community history. The first treatment subplot was one with soil history but without plant history: After removing the existing plant community as well as the upper ~2 cm of soil containing the plot seed bank, the soil was mixed to 30 cm depth. The subplot was then re‐sown with the same plant species as done in 2002. The second treatment subplot was one with neither soil nor plant history: Soil was excavated to a depth of 30 cm, replaced with soil from an arable field and re‐sown with the same plant species.

### 2.3 Sampling and data collection

The sampling campaign took place between 14 and 24 June 2021, shortly after the first plant biomass harvest and at peak biological activity. From each subplot, we extracted one soil core of 20 cm Ø, one soil core of 5 cm Ø and four cores of 2 cm Ø. The sampled depth was 10 cm for all cores.

For each subplot, we pooled the 2 cm Ø cores and then sieved the soil to break large aggregates and removed seeds and roots. To assess nematode taxa composition and density, we extracted nematodes from ~25 g of the sieved soil, using a modified Baermann–Funnel method (Cesarz et al., 2019). We then counted the extracted individuals and randomly identified up to 100 individuals from each sample to genus or family level. The density of nematodes per m^2^ was estimated based on the number of individuals per gram of dry soil and the gram of dry soil per cm^3^ (i.e. we calculated the number of individuals of each nematode taxon in a volume of 100 × 100 × 10 cm). The taxon composition of the identified subsample was then extrapolated to the estimated density of nematodes per m^2^.

Macrofauna were extracted by heat from the 20 cm Ø cores (Kempson et al., 1963), while mesofauna were extracted from the 5 cm Ø cores (Macfadyen, 1961). To extract soil mesofauna, we split the 10 cm soil core into 5 cm cores, to increase extraction efficiency. The animals extracted were stored in 65% ethanol. Mesofauna were sorted to Acari, Collembola, Protura, Pauropoda and Symphyla, and subsequently Acari and Collembola were identified to order and family level, respectively. Macrofauna were identified to order level. To calculate the density of macrofauna and mesofauna taxa, we extrapolated from the number of individuals found within the surface sampled by the respective core to the number of individuals per m^2^. The loss of vials during processing resulted in lack of information for Acari and Collembola in 7 out of 240 subplots. We used multiple imputation of missing data as implemented by the *mice* package (Buuren & Groothuis‐Oudshoorn, 2011) to impute the density of the different Acari and Collembola groups in the samples that lacked this information. This resulted in 100 versions of the subplot by taxon dataframe, capturing uncertainty for the imputed values. This approach allowed us to estimate energy fluxes for all 240 subplots.

Our study captures a considerable portion of the soil fauna community, with all its trophic functions well represented (by herbivores i.e. root feeders, predators, primary and secondary decomposers). We will subsequently refer to the soil fauna community, acknowledging that we are dealing with a representative and consistent subset of it.

This study did not require ethical approval.

### 2.4 Calculation of energy flux

**FIGURE 1:**
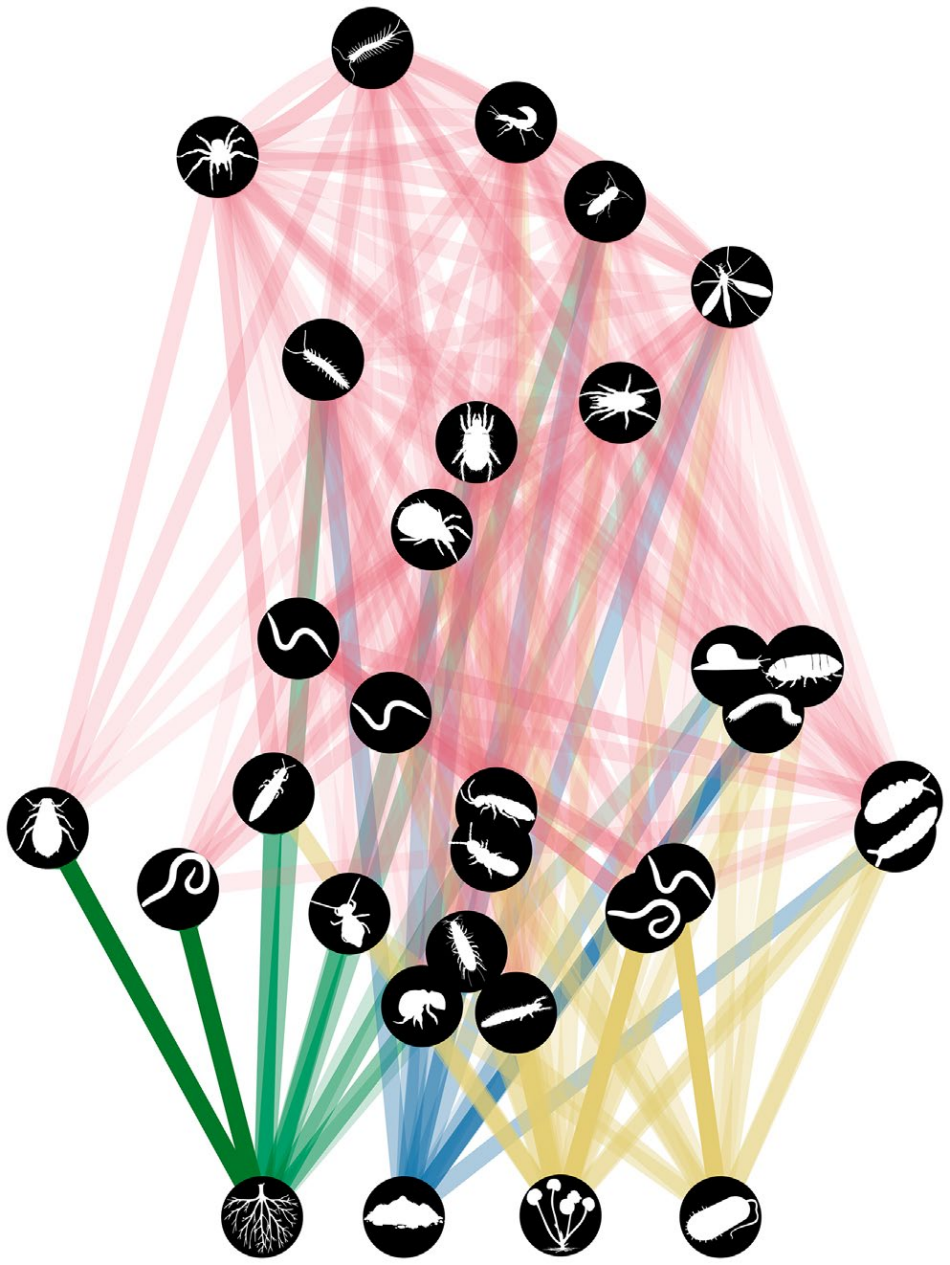
The meta food web of the soil fauna community, depicting predatory (red), herbivorous (green), detritivorous (blue) and microbivorous (yellow) interactions of the taxa listed in Table S2. Silhouettes are public domain or CC‐BY 3.0 and available via phylopic.org (details in Table S3).

We calculated energy flux for each of the 240 soil food webs in the Jena Experiment using the *fluxweb* package (Gauzens, 2018; Gauzens et al., 2019). Details on the concept and application of this framework can be found in Barnes et al. (2018) and Jochum et al. (2021). Briefly, the energy that flows across every link in a food web is inferred by considering energetic losses of each node due to metabolism and consumption. That is, under a steady‐state assumption, every node (population) is compensating its losses by absorbing energy from its resources. Due to assimilation inefficiencies, a surplus of energy is required to compensate for a given amount of lost energy. Fluxes are calculated from the top to the bottom of the food web, so the energy that flows out of a trophic level is enough to support all the levels above it (Figure 1).

### 2.5 Population level metabolic losses

Resting metabolic rate is a power‐law function of body mass (Ehnes et al., 2011). To estimate the body mass distribution of the different taxa, we aimed to measure the length (and width in the case of macrofauna) of up to 10 individuals per taxon per subplot. As the large number of samples made it infeasible to do this for all subplots, we did so selectively for up to 24 samples spanning the plant richness gradient. We then used published taxon‐specific relationships of length (and width) to mass (Mercer et al., 2001; Sohlström et al., 2018) to calculate the body mass of each measured individual. By pooling information across samples, we determined the body mass distribution characteristic of each taxon, expressed by its mean and standard deviation. To estimate population level metabolic losses per m^2^, we first drew *N* samples from lognormal distribution based on the calculated mean and sd, where *N* is the number of individuals/m^2^ of a given taxon. We then calculated metabolic losses as a function of body mass (based on Ehnes et al. (2011)) for the *N* body masses and summed them up to population level losses.

### 2.6 The trophic interaction matrix

We used information on the trophic relationships of the different soil fauna groups (as reviewed in Potapov et al. (2022)) as well as traits that influence the strength of these interactions (Potapov, 2022), combined with our data of the biomass and body mass distribution of the different taxa, to estimate energy fluxes in the soil food web (Barnes et al., 2018; Jochum et al., 2021; Potapov, 2022). For the feeding type and body mass distributions of the different nematode taxa we relied on Nemaplex (Ferris, 1999). Collembola were grouped to functional leagues according to Potapov et al. (2016).

We started by constructing a square matrix m expressing trophic relationships among all trophic groups observed in the entire experiment, as well as four basal resources (roots, detritus, bacteria and fungi). When taxon i is consumed by taxon j, $m_{\mathrm{ij}}$ has a non‐zero value. Initial values were chosen to reflect broad preferences of the different trophic groups (Potapov et al., 2022). For example, Diplopoda are primarily detritivores that also consume microbes. This can be expressed as an expected diet composition of 75% detritus and 12.5% each for fungi and bacteria. In the case of predatory interactions, to begin with, we used values reflecting equal preference among potential prey. Once this preliminary matrix was complete (Table S1), we used additional information such as predator–prey body mass ratios (Brose et al., 2006) as well as prey attributes such as agility or the possession of physical or chemical defences and finally, the probability of encounter between individuals of different taxa given their similarity in vertical stratification, to refine the expected interaction strength among taxa (following Potapov, 2022). At this stage, the matrix expressed the expected affinity for different resources.

This matrix was subsequently split into 240 subplot‐specific matrices, containing only the basal resources and the taxa found in each subplot. Then, trophic interactions were further modified by the relative availability of different prey taxa (based on relative biomass). Therefore, the elements of each column in the resulting matrices expressed the expected diet composition of each consumer j. The matrix elements are a composite of probability of encounter, probability of a predator of certain size to subdue prey of a certain size or with certain physical or chemical attributes. Accordingly, to account for the inherently probabilistic nature of these interactions and allow for some diet uncertainty, we treated the elements in each matrix column as the component probabilities of a Dirichlet distribution (see Figure S2 and accompanying text). We generated 1000 versions of each subplot‐specific matrix; in each version, the elements of each column were one sample from a Dirichlet distribution whose component probabilities was the vector of the original elements, multiplied by a constant. In practice, this meant that zero elements remained zero and non‐zero elements were approximately normally distributed around the expected value, while column sums were constrained to 1. Therefore, a consumer's diet was, on average, the expected diet but with some variation around this expectation. The amount of variation depends on the constant (higher values result in less variation). We tested the sensitivity of our energy flux estimates and any subsequent inferences by choosing different levels of the constant (Figure S2). Combining the 1000 matrices with the multiple imputation described above, our modified application of this framework accounts for the uncertainty of trophic interactions as well as uncertainty for the missing data. Due to the probabilistic nature of our interaction matrix, the estimated energy fluxes were also distributions rather than single values.

### 2.7 Community level energy flux

We calculated the total energy flux in the soil fauna community by summing the energy of all individual links in each food web. This quantity is a measure of the composite multitrophic functioning of the soil fauna community. We additionally calculated the sum of energy flux of links that correspond to distinct trophic functions, namely herbivory, predation, detritivory as well as microbivory.

### 2.8 Below‐ground herbivory pressure

We calculated herbivory pressure as the sum of outflux of energy from plants to their consumers (including omnivores) per mg of root biomass. Root biomass data were available for a 0–5 cm depth across all experimental units (data for 5–10 cm were only available for the control subplots) while energy fluxes were based on animals sampled at a 0–10 cm depth. We have conducted a sensitivity analysis to test the influence of excluding the 5–10 cm layer in control subplots.

### 2.9 Control of herbivory

In the absence of omnivores, control of herbivory through predation can be quantified as the ratio of outfluxes from herbivores to their consumers over the influxes to herbivores (outfluxes from plants to herbivores times assimilation efficiency). Given the steady‐state assumption, this quantity is a fraction, expressing how much of the energy that is absorbed by herbivores is taken away from them through consumption. Omnivores complicate this calculation, as their outfluxes are partly relevant for herbivory control but only to the extent that omnivores rely on plants. To incorporate omnivores in the calculation of herbivory control, the numerator was instead the sum of outfluxes from plant consumers after those had been weighted by each consumer's proportion of energy uptake that comes from plants (1 for herbivores, <1 for omnivores). The denominator was the sum of energy influxes from plants to plant consumers.

### 2.10 Statistical analysis

We examined whether the relationship between plant species richness and the energy flows of interest (community level, herbivory pressure and control, detritivory and microbivory) differs depending on the absence versus presence of plant and soil history. To get a better understanding of any effects on the fluxes of interest, we conducted additional analyses with overall predation and overall herbivory as a response. Our models had the general formula

$$\begin{array}{c} \text{response}.\text{mean}|\mathrm{mi}\left(\text{response}.\mathrm{sd}\right)\\ \sim 1+\text{richness}\times \text{history}+\left(1|\text{block}/\text{plot}\right). \end{array}$$

The left‐hand side of the formula indicates that the response consists of distributions rather than single values, defined by the mean and the standard deviation of the energy flux across the 1000 versions of each food web. This distribution reflects the uncertainty for the real value. We, therefore, employ an analytical approach that is used to account for measurement error (Bürkner, 2021; McElreath, 2020) to incorporate the varying flux uncertainty that was produced by diet composition uncertainty (Figures S2 and S3). After an initial modelling attempt, posterior predictive checks showed that linear models failed to reproduce the right skewed distribution of observed values. We therefore log‐transformed fluxes before calculating the mean and sd across the 1000 versions. The exception to this was herbivory control which, as a continuous proportion, was modelled with a Beta distribution.

The right‐hand side of the formula indicates that we are estimating the coefficients for the intercept (*1*) and slope (richness) of the average relationship between response and plant species richness for the control subplots, and the coefficients for the difference in intercept and slope between each treatment and control, accounting for treatment subplots being nested within plots, which themselves are nested within blocks. Plant species richness was log‐transformed (base 2), centred and scaled.

We fitted models in Stan (Stan Development Team, 2024, CmdStan v.2.35) via the *brms* package (Bürkner, 2018, v.2.19.0), using default priors and four MCMC chains with at least 4000 iterations each (with the first half used for warm‐up). We evaluated our models with posterior predictive checks, visual inspection of chain mixing, as well as Rhat values (not exceeding 1.01).

We report mean estimates and 90% highest posterior density intervals (HPD) of slopes and their contrasts, extracted using the *emmeans* package (Lenth, 2023, *v*.1.8.6). We note the sign of a relationship and use the exclusion of zero from the interval to evaluate whether a relationship is statistically clear or not (Dushoff et al., 2019).

Finally, we examined the sensitivity of our results on assuming different levels of diet uncertainty by repeating our analyses for three levels of uncertainty as well as without uncertainty (results reported in the main text are for intermediate uncertainty). We found that the coefficients of our models were robust to increasing diet uncertainty (Figure S4).

## 3 RESULTS

### 3.1 Community‐level energy flux

**FIGURE 2:**
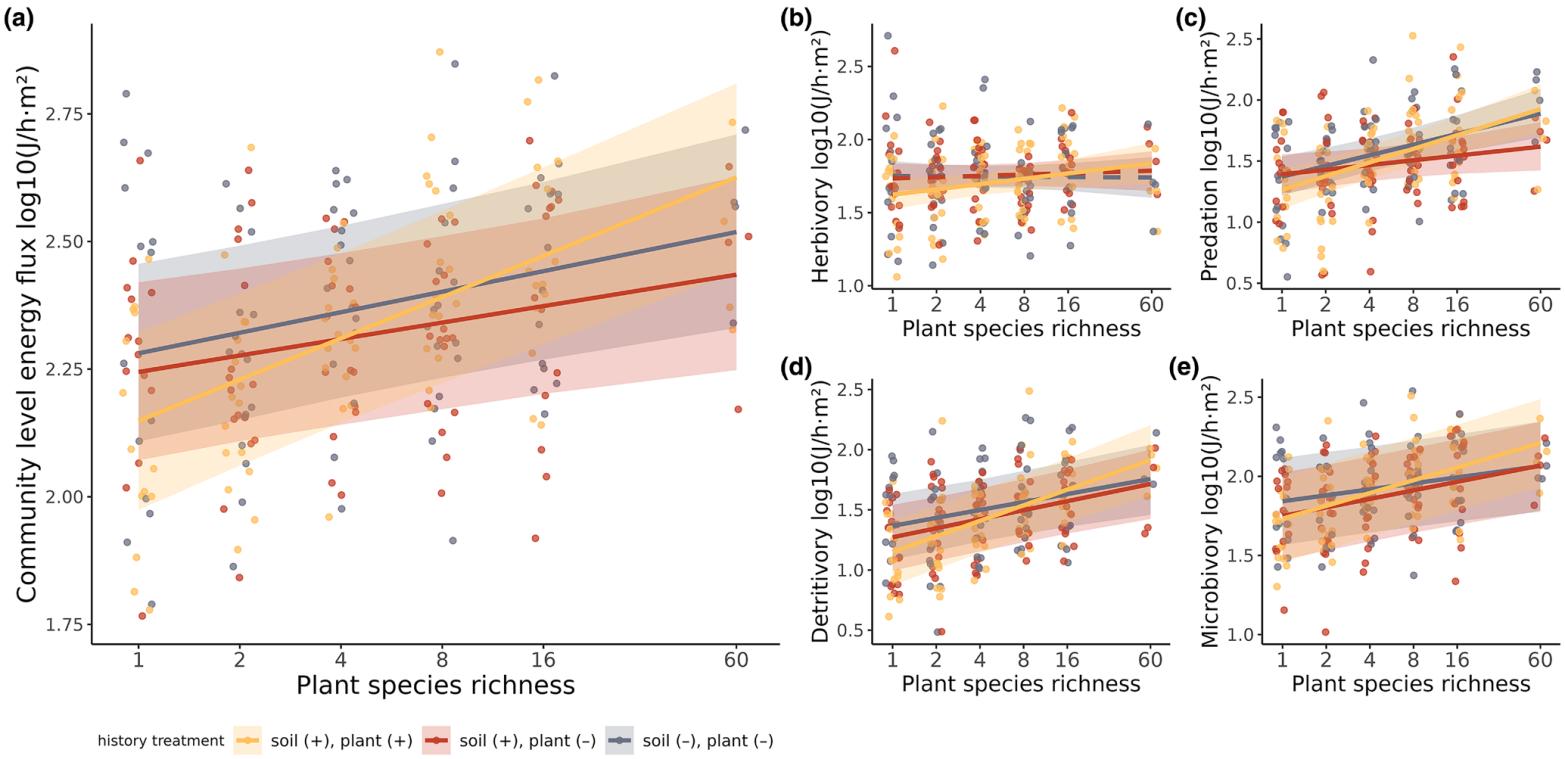
The relationship between plant richness and summed energy flux for different combinations of plant and soil community history. (a) Total energy flux, (b) herbivory fluxes, (c) predation, (d) detritivory and (e) microbivory. Lines show mean estimates for the average richness–flux relationship bound by 90% uncertainty intervals. Dashed lines indicate relationships whose slope is not clearly different from zero.

Plant richness had a clear positive effect on community‐level flux, in control communities with plant and soil history (mean slope [90% HPD] = 0.13 [0.09, 0.16], Figure 2a). As expected, this relationship was shallower in the case of the treatment communities lacking aspects of shared history (with soil but not plant history: 0.05 [0.02, 0.09]; without soil or plant history: 0.06 [0.03, 0.10]). In both cases, the difference between the slope in control and that in treatment communities was clear (−0.08 [−0.12, −0.03] and −0.06 [−0.11, −0.02], respectively, Figure 2a).

### 3.2 Individual trophic functions

Plant richness had a weakly positive but clear effect on herbivory in communities with soil and plant history (0.05 [0.01, 0.105], Figure 2b). This relationship was weakly positive or negative but very unclear for the two history treatments (with soil but not plant history: 0.01 [−0.035, 0.06]; without soil or plant history: −0.003 [−0.05, 0.04], Figure 2b).

The effect of plant richness on predation was clearly positive across control and treatment communities (with soil and plant history: 0.18 [0.12, 0.24]; with soil but not plant history: 0.06 [0.003, 0.12]; without soil or plant history: 0.14 [0.08, 0.20], Figure 2c). The slope of the relationship in the case of soil but no plant history was shallowest and clearly different from that of control communities (−0.12 [−0.18, −0.05]), while the relationship across communities without soil or plant history was intermediate but still clearly different from the other treatments (0.08 [0.02, 0.15]).

The effect of plant richness on detritivory was clearly positive across control and treatment communities (with soil and plant history: 0.21 [0.15, 0.265]; with soil but not plant history: 0.12 [0.06, 0.17]; without soil or plant history 0.11 [0.05, 0.16], Figure 2d). The slope was steeper across communities with soil and plant history and clearly different from the two treatments (0.08 [0.02, 0.16] and 0.10 [0.03, 0.18]). The effect of plant richness on microbivory was also positive (with soil and plant history: 0.13 [0.09, 0.17]; with soil but not plant history: 0.09 [0.05, 0.12]; without soil or plant history 0.06 [0.02, 0.10], Figure 2e). Once again, the slope was steeper across communities with soil and plant history compared to the two treatments, but this difference was only clear when compared to communities without soil or plant history (−0.07 [−0.12, −0.02]).

### 3.3 Herbivory pressure on plants and control of herbivory by predation

**FIGURE 3:**
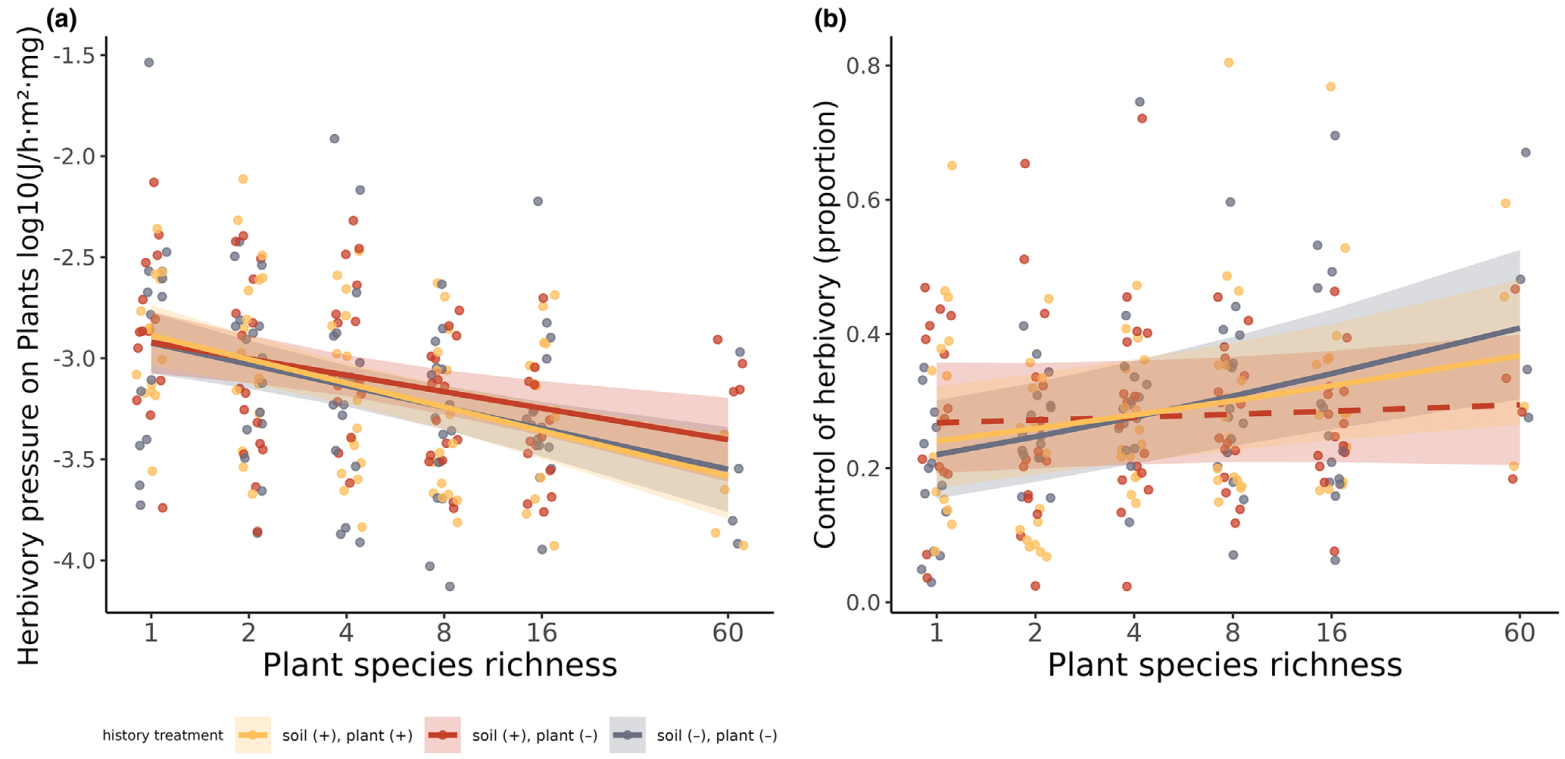
The relationship between plant richness and (a) herbivory pressure on plants and (b) control of herbivory through predation. Lines show mean estimates for the average richness–function relationship bound by 90% uncertainty intervals. Dashed lines indicate relationships whose slope is not clearly different from zero.

Plant‐rich communities experienced reduced herbivory pressure (with soil and plant history: −0.19 [−0.265, −0.11]; with soil but not plant history: −0.13 [−0.21, −0.05]; without soil or plant history −0.17 [−0.25, −0.09], Figure 3a). There were no clear differences in slope between control and treatments for any pairwise combination. In a sensitivity analysis, this negative relationship between herbivory pressure and plant species richness was robust to increasing root measurement depth (Figure S6). Finally, the relationship of herbivory control through predation with plant richness was clearly positive only in control communities and those without soil or plant history (with soil and plant history: 0.17 [0.04, 0.28]; with soil but not plant history: 0.035 [−0.08, 0.16]; without soil or plant history: 0.24 [0.12, 0.36]). Only the richness–control relationship across communities without soil or plant history was clearly stronger than that across communities with soil but no plant history (0.21 [0.06, 0.37]).

## 4 DISCUSSION

In our study manipulating plant species richness across treatments of soil and plant community history, we found that plant‐rich communities support higher levels of multitrophic functioning of the soil fauna community. Moreover, we found that this diversity–function relationship was generally weaker in communities without shared community history, with only minor differences between the two history removal treatments. Together, these results confirm that the steepening of the diversity functioning relationship is driven by processes that emerge during community development.

Here, we provide experimental evidence of a positive effect of plant richness on the functioning of the soil fauna food web. Previous studies have demonstrated that plant diversity has a positive effect on the abundance and diversity of the invertebrate community, below as well as above‐ground (Ebeling et al., 2018; Milcu et al., 2013; Scherber et al., 2010), suggesting consequent changes on their ecosystem functioning. Subsequent research has corroborated the relationship between diversity and invertebrate food web functioning above‐ground (Barnes et al., 2020; Buzhdygan et al., 2020). However, evidence of a link between plant diversity and soil fauna functions has remained elusive (Buzhdygan et al., 2020). A particular challenge of the below‐ground component of an ecosystem is that, with the exception of detritivory (Birkhofer et al., 2011), the feeding activity of soil fauna is difficult to assess directly. The calculation of energy flux in a food web provides a way to circumvent this limitation. This method relies on existing knowledge of the trophic ecology of soil fauna (as reviewed by Potapov et al., 2022) and is inevitably an approximation. However, here we have improved its application by incorporating diet uncertainty in the calculation of energy fluxes.

Our findings show that indeed the soil fauna community multitrophic functioning, estimated by the overall energy that flows across links in the soil food web, increases with increasing plant richness. When considering trophic functions separately, we found that this relationship is stronger for the brown food web (detritivory, microbivory) and predation, while the effect of plant richness on herbivory was weaker and context dependent. Our approach of applying energy flux calculations to below‐ground food webs has thus demonstrated that plant diversity has a positive effect on functioning, despite some variation across different ecosystem functions.

The evidence of a positive effect of plant richness on invertebrate communities and their functioning comes from data that were collected some years after the establishment of an experiment (Barnes et al., 2020; Buzhdygan et al., 2020; Ebeling et al., 2018; Milcu et al., 2013; Scherber et al., 2010) or in unmanipulated ecosystems (Birkhofer et al., 2011). In other words, these relationships have generally been observed in established communities with a shared history among producers and consumers. At the same time, longitudinal data of other ecosystem functions, such as primary productivity (Huang et al., 2018; Reich et al., 2012) or soil microbial activity (Eisenhauer et al., 2010), have shown that BEF relationships may be absent or weak in the early stages of a community's development and emerge or become stronger later on. This has led to our hypothesis that disrupting the biotic interactions that have been formed during a community's history by experimentally removing components of this history would diminish the positive effect of plant diversity on soil fauna community functioning. Our results largely support this hypothesis; the relationship of community‐level energy flux was clearly stronger across control communities with both soil and plant community history, compared to either of the two history removal treatments. The removal of plant history was apparently enough to diminish the overall BEF relationship as the two treatments had a similar pattern. This effect may be attributed to the absence of plant adaptations to the soil microbial and faunal community (Dorey et al., 2024; Semchenko et al., 2019) across all communities without plant history. Alternatively, it could arise from the disruption of plant‐related soil community attributes, such as mycorrhizal associations (Albracht et al., 2024; Hahl et al., 2020) or the disturbance of the rhizosphere soil community more generally. Although the difference to the two treatments was not always clear, the slope of the diversity–function relationship was consistently steeper among control communities, regardless of the specific trophic function considered. In the case of trophic functions of the brown food web, the most pronounced difference of control communities was with communities with neither soil nor plant history, which exhibited the shallowest BEF relationships. However, contrary to our hypothesis, the diversity–predation relationship was weakest across communities with soil but not plant history, with no‐history communities having a relationship similar to control ones. This suggests that the mismatch between soil and plant history might be more detrimental for some processes. The plant community itself should be most sensitive to this mismatch (Semchenko et al., 2019; Van Der Putten et al., 2013), with ‘naive’ plant communities exposed to an established community of plant antagonists, but this was not reflected in the diversity–root biomass relationship. Predators are subsidized by lower level consumers, but the effects of diversity on energy flows to those levels across treatments also cannot account for this difference. Overall, our findings indicate that biotic changes that take place in communities over time are responsible for the strengthening of BEF relationships below‐ground.

We also considered the effect of plant diversity on herbivory pressure on plants, as well as herbivory control through predation. The relationship of these functions to plant diversity has been examined in above‐ground consumers of well‐established communities (Barnes et al., 2020; Ristok et al., 2023). In such mature communities, control of herbivory was shown to increase with plant richness, while herbivory pressure had the opposite relationship with plant richness, indicating a top‐down mechanism (Barnes et al., 2020). Here, we hypothesized this mechanism to be emerging through the restructuring of trophic interactions over time. We did find evidence of increasing herbivory control with increasing richness, across control communities and across communities without any history but not in those of the intermediate treatment, which partly reflects what we found for overall predation. At the same time, herbivory pressure was indeed reduced with increasing plant richness, with no clear effects of community history on the strength of this relationship. This reduction of pressure seems to emerge from weakly increasing or unchanging herbivory, combined with a clear increase of root biomass with increasing plant richness (Figure S5). The fact that herbivory pressure was reduced with higher richness even without a corresponding increase of herbivory control (as was the case across communities with soil but not plant history) indicates that the latter was not crucial for this reduction. We, therefore, suggest the presence of an alternative mechanism for the multitrophic reduction of herbivory pressure: the functioning of the brown food web, which is instrumental for nutrient availability (Wardle et al., 2004; Wurst, 2013), was positively influenced by plant richness. This relationship can in turn enhance plant productivity in plant‐rich communities, leading to the observed net reduction of herbivory pressure. Therefore, different multitrophic mechanisms can be important for promoting plant productivity, depending on whether we consider the above‐ or below‐ground component of an ecosystem (Barnes et al., 2020; Jochum et al., 2021; Ristok et al., 2023).

Our findings indicate that the effects of biodiversity on below‐ground ecosystem functioning are dependent on the shared history of producers and consumers in the community, supporting the idea that a combination of niche differentiation with turnover processes is reshaping this relationship over time. This suggests that BEF relationships are context dependent, varying not only across space (Thompson et al., 2018) but also in time. Our study contributes to experimental evidence (Schmid et al., 2021; van Moorsel et al., 2020; Zuppinger‐Dingley et al., 2015) that the directional temporal change of BEF relationships that has been observed in long‐term experiments (Huang et al., 2018; Reich et al., 2012; Wagg et al., 2022) is indeed related to community development and highlights the role of below‐ground consumers. In natural ecosystems, the trajectory of community change over time will likely be influenced by factors that determine the potential for plant niche differentiation but also those regulating animal community assembly, such as latitudinal or environmental gradients of regional species richness or landscape characteristics that affect accessibility through dispersal (Ye & Wang, 2023). Therefore, to understand how BEF relationships develop over time, future research should traverse the temporal and spatial dimension, examining how meta‐community processes shape local dynamics (Amarasekare, 2008; Fletcher et al., 2024).

## AUTHOR CONTRIBUTIONS

Ulrich Brose, Nico Eisenhauer and Stefan Scheu conceived the idea of the study and acquired funding. Angelos Amyntas conducted the sampling. Marcel Ciobanu, Lara Warnke, Mark Maraun, Jörg‐Alfred Salamon, Mona Merkle identified soil fauna. Leonardo Bassi, Justus Hennecke, Markus Lange and Gerd Gleixner provided root and soil data. Angelos Amyntas performed the food web construction and calculations with input from Benoit Gauzens and conducted the statistical analysis. Angelos Amyntas wrote the first draft of the manuscript with support from Ulrich Brose. All authors contributed to revisions.

## CONFLICT OF INTEREST STATEMENT

The authors declare that they have no conflicts of interest.

## Supporting information


**Table S1:** The initial interaction matrix, before considering traits, reflecting what the different taxa feed on, as reviewed in Potapov et al. (2022).
**Table S2:** The attributes of taxa that co‐determine the relative strengths in the interaction matrix.
**Tables S3–S4:** Information on the creators and license of the silhouettes used in Figure 1 of the main text.
**Figure S1:** Schematic representation of the calculation of the probability of a predator consuming certain prey taxa based on body‐mass, using Araneae and Hemiptera as an example.
**Figure S2:** Example of low (top), intermediate (middle) and high (bottom) consumer diet uncertainty, for a hypothetical consumer with an expected diet composition of (0.1, 0.3, 0.6) of three prey taxa.
**Figure S3:** The effect of low (top), intermediate (middle) and high (bottom) consumer diet uncertainty on community level energy flux.
**Figure S4:** Model coefficients excluding diet uncertainty (‐) and at low (1000), intermediate (100) and high (10) uncertainty.
**Figure S5:** The relationship between plant richness and root biomass in the 0‐5 cm depth soil layer.
**Figure S6:** The relationship of plant richness and root biomass in the 0‐10 cm depth soil layer in control subplots (left).

## Data Availability

The data and code necessary to reproduce the calculations of fluxes, the analyses and figures are available on Github (https://github.com/amynang/dBEF_soil_foodwebs_jae) and have been archived in Zenodo (https://doi.org/10.5281/zenodo.14513121; Amyntas et al., 2024).
