# Supplementary material for: Shared community history strengthens plant diversity effects on below‐ground multitrophic functioning
Source: J Anim Ecol. 2025 Jan 29;94(4):555–65. doi: 10.1111/1365-2656.14241 (PMC11962227; doi:10.1111/1365-2656.14241)
Supplement: Supplementary file 1 — Table S1: The initial interaction matrix, before considering traits, reflecting what the different taxa feed on, as reviewed in Potapov et al. (2022). Table S2: The attributes of taxa that co‐determine the relative strengths in the interaction matrix. Tables S3–S4: Information on the creators and license of the silhouettes used in Figure 1 of the main text. Figure S1: Schematic representation of the calculation of the probability of a predator consuming certain prey taxa based on body‐mass, using Araneae and Hemiptera as an example. Figure S2: Example of low (top), intermediate (middle) and high (bottom) consumer diet uncertainty, for a hypothetical consumer with an expected diet composition of (0.1, 0.3, 0.6) of three prey taxa. Figure S3: The effect of low (top), intermediate (middle) and high (bottom) consumer diet uncertainty on community level energy flux. Figure S4: Model coefficients excluding diet uncertainty (‐) and at low (1000), intermediate (100) and high (10) uncertainty. Figure S5: The relationship between plant richness and root biomass in the 0‐5 cm depth soil layer. Figure S6: The relationship of plant richness and root biomass in the 0‐10 cm depth soil layer in control subplots (left). [file JANE-94-555-s001.docx]

**Table S1**. The initial interaction matrix, before considering traits, reflecting what the different taxa feed on, as reviewed in Potapov et al. (2022). Unless more detailed information was available, omnivores were assumed to feed equally from different resource channels (eg. roots, detritus, animal prey). Values shown here have been rounded to 2 significant digits

|  | Bacterivore.nematodes | Fungivore.nematodes | Herbivore.nematodes | Omnivore.nematodes | Predator.nematodes | Edaphic.Entomobryomorpha | Edaphic.Neelipleona | Edaphic.Poduromorpha | Epigeic.Entomobryomorpha | Epigeic.Poduromorpha | Epigeic.Symphypleona | Mesostigmata | Oribatida | Pauropoda | Prostigmata | Protura | Symphyla | Araneae | Chilopoda | Coleoptera | Staphylinidae | Diplopoda | Gastropoda | Hemiptera | Isopoda | Thysanoptera | Diptera.larvae |
| --- | --- | --- | --- | --- | --- | --- | --- | --- | --- | --- | --- | --- | --- | --- | --- | --- | --- | --- | --- | --- | --- | --- | --- | --- | --- | --- | --- |
| roots | 0 | 0 | 1 | 0 | 0 | 0 | 0 | 0 | 0.33 | 0 | 0.33 | 0 | 0 | 0.33 | 0.25 | 0 | 0.33 | 0 | 0 | 0.25 | 0 | 0 | 0.1 | 1 | 0 | 0.5 | 0.1 |
| detritus | 0 | 0 | 0 | 0 | 0 | 0.33 | 0.33 | 0.33 | 0 | 0 |  | 0 | 0.25 | 0.33 | 0.25 | 0.1 | 0.33 | 0 | 0 | 0.25 | 0 | 0.75 | 0.3 | 0 | 0.33 | 0 | 0.3 |
| bacteria | 1 | 0 | 0 | 0.25 | 0 | 0.33 | 0.33 | 0.33 | 0.33 | 0.33 | 0.33 | 0 | 0.25 | 0 | 0 | 0 | 0 | 0 | 0 | 0 | 0 | 0.13 | 0.3 | 0 | 0.33 | 0 | 0 |
| fungi | 0 | 1 | 0 | 0.25 | 0 | 0.33 | 0.33 | 0.33 | 0.33 | 0.33 | 0.33 | 0 | 0.25 | 0.33 | 0.25 | 0.9 | 0 | 0 | 0 | 0.25 | 0.2 | 0.13 | 0.3 | 0 | 0.33 | 0.5 | 0.3 |
| Bacterivore.nematodes | 0 | 0 | 0 | 0.1 | 0.2 | 0 | 0 | 0 | 0 | 0.07 | 0 | 0.06 | 0.05 | 0 | 0.01 | 0 | 0.02 | 0 | 0 | 0 | 0 | 0 | 0 | 0 | 0 | 0 | 0.01 |
| Fungivore.nematodes | 0 | 0 | 0 | 0.1 | 0.2 | 0 | 0 | 0 | 0 | 0.07 | 0 | 0.06 | 0.05 | 0 | 0.01 | 0 | 0.02 | 0 | 0 | 0 | 0 | 0 | 0 | 0 | 0 | 0 | 0.01 |
| Herbivore.nematodes | 0 | 0 | 0 | 0.1 | 0.2 | 0 | 0 | 0 | 0 | 0.07 | 0 | 0.06 | 0.05 | 0 | 0.01 | 0 | 0.02 | 0 | 0 | 0 | 0 | 0 | 0 | 0 | 0 | 0 | 0.01 |
| Omnivore.nematodes | 0 | 0 | 0 | 0.1 | 0.2 | 0 | 0 | 0 | 0 | 0.07 | 0 | 0.06 | 0.05 | 0 | 0.01 | 0 | 0.02 | 0 | 0 | 0 | 0 | 0 | 0 | 0 | 0 | 0 | 0.01 |
| Predator.nematodes | 0 | 0 | 0 | 0.1 | 0.2 | 0 | 0 | 0 | 0 | 0.07 | 0 | 0.06 | 0.05 | 0 | 0.01 | 0 | 0.02 | 0 | 0 | 0 | 0 | 0 | 0 | 0 | 0 | 0 | 0.01 |
| Edaphic.Entomobryomorpha | 0 | 0 | 0 | 0 | 0 | 0 | 0 | 0 | 0 | 0 | 0 | 0.06 | 0 | 0 | 0.01 | 0 | 0.02 | 0.05 | 0.05 | 0.01 | 0.04 | 0 | 0 | 0 | 0 | 0 | 0.01 |
| Edaphic.Neelipleona | 0 | 0 | 0 | 0 | 0 | 0 | 0 | 0 | 0 | 0 | 0 | 0.06 | 0 | 0 | 0.01 | 0 | 0.02 | 0.05 | 0.05 | 0.01 | 0.04 | 0 | 0 | 0 | 0 | 0 | 0.01 |
| Edaphic.Poduromorpha | 0 | 0 | 0 | 0 | 0 | 0 | 0 | 0 | 0 | 0 | 0 | 0.06 | 0 | 0 | 0.01 | 0 | 0.02 | 0.05 | 0.05 | 0.01 | 0.04 | 0 | 0 | 0 | 0 | 0 | 0.01 |
| Epigeic.Entomobryomorpha | 0 | 0 | 0 | 0 | 0 | 0 | 0 | 0 | 0 | 0 | 0 | 0.06 | 0 | 0 | 0.01 | 0 | 0.02 | 0.05 | 0.05 | 0.01 | 0.04 | 0 | 0 | 0 | 0 | 0 | 0.01 |
| Epigeic.Poduromorpha | 0 | 0 | 0 | 0 | 0 | 0 | 0 | 0 | 0 | 0 | 0 | 0.06 | 0 | 0 | 0.01 | 0 | 0.02 | 0.05 | 0.05 | 0.01 | 0.04 | 0 | 0 | 0 | 0 | 0 | 0.01 |
| Epigeic.Symphypleona | 0 | 0 | 0 | 0 | 0 | 0 | 0 | 0 | 0 | 0 | 0 | 0.06 | 0 | 0 | 0.01 | 0 | 0.02 | 0.05 | 0.05 | 0.01 | 0.04 | 0 | 0 | 0 | 0 | 0 | 0.01 |
| Mesostigmata | 0 | 0 | 0 | 0 | 0 | 0 | 0 | 0 | 0 | 0 | 0 | 0.06 | 0 | 0 | 0.01 | 0 | 0.02 | 0.05 | 0.05 | 0.01 | 0.04 | 0 | 0 | 0 | 0 | 0 | 0.01 |
| Oribatida | 0 | 0 | 0 | 0 | 0 | 0 | 0 | 0 | 0 | 0 | 0 | 0.06 | 0 | 0 | 0.01 | 0 | 0.02 | 0.05 | 0.05 | 0.01 | 0.04 | 0 | 0 | 0 | 0 | 0 | 0.01 |
| Pauropoda | 0 | 0 | 0 | 0 | 0 | 0 | 0 | 0 | 0 | 0 | 0 | 0.06 | 0 | 0 | 0.01 | 0 | 0.02 | 0.05 | 0.05 | 0.01 | 0.04 | 0 | 0 | 0 | 0 | 0 | 0.01 |
| Prostigmata | 0 | 0 | 0 | 0 | 0 | 0 | 0 | 0 | 0 | 0 | 0 | 0.06 | 0 | 0 | 0.01 | 0 | 0.02 | 0.05 | 0.05 | 0.01 | 0.04 | 0 | 0 | 0 | 0 | 0 | 0.01 |
| Protura | 0 | 0 | 0 | 0 | 0 | 0 | 0 | 0 | 0 | 0 | 0 | 0.06 | 0 | 0 | 0.01 | 0 | 0.02 | 0.05 | 0.05 | 0.01 | 0.04 | 0 | 0 | 0 | 0 | 0 | 0.01 |
| Symphyla | 0 | 0 | 0 | 0 | 0 | 0 | 0 | 0 | 0 | 0 | 0 | 0.06 | 0 | 0 | 0.01 | 0 | 0.02 | 0.05 | 0.05 | 0.01 | 0.04 | 0 | 0 | 0 | 0 | 0 | 0.01 |
| Araneae | 0 | 0 | 0 | 0 | 0 | 0 | 0 | 0 | 0 | 0 | 0 | 0 | 0 | 0 | 0 | 0 | 0 | 0.05 | 0.05 | 0.01 | 0.04 | 0 | 0 | 0 | 0 | 0 | 0.01 |
| Chilopoda | 0 | 0 | 0 | 0 | 0 | 0 | 0 | 0 | 0 | 0 | 0 | 0 | 0 | 0 | 0 | 0 | 0 | 0.05 | 0.05 | 0.01 | 0.04 | 0 | 0 | 0 | 0 | 0 | 0.01 |
| Coleoptera | 0 | 0 | 0 | 0 | 0 | 0 | 0 | 0 | 0 | 0 | 0 | 0 | 0 | 0 | 0 | 0 | 0 | 0.05 | 0.05 | 0.01 | 0.04 | 0 | 0 | 0 | 0 | 0 | 0.01 |
| Staphylinidae | 0 | 0 | 0 | 0 | 0 | 0 | 0 | 0 | 0 | 0 | 0 | 0 | 0 | 0 | 0 | 0 | 0 | 0.05 | 0.05 | 0.01 | 0.04 | 0 | 0 | 0 | 0 | 0 | 0.01 |
| Diplopoda | 0 | 0 | 0 | 0 | 0 | 0 | 0 | 0 | 0 | 0 | 0 | 0 | 0 | 0 | 0 | 0 | 0 | 0.05 | 0.05 | 0.01 | 0.04 | 0 | 0 | 0 | 0 | 0 | 0.01 |
| Gastropoda | 0 | 0 | 0 | 0 | 0 | 0 | 0 | 0 | 0 | 0 | 0 | 0 | 0 | 0 | 0 | 0 | 0 | 0.05 | 0.05 | 0.01 | 0.04 | 0 | 0 | 0 | 0 | 0 | 0.01 |
| Hemiptera | 0 | 0 | 0 | 0 | 0 | 0 | 0 | 0 | 0 | 0 | 0 | 0 | 0 | 0 | 0 | 0 | 0 | 0.05 | 0.05 | 0.01 | 0.04 | 0 | 0 | 0 | 0 | 0 | 0.01 |
| Isopoda | 0 | 0 | 0 | 0 | 0 | 0 | 0 | 0 | 0 | 0 | 0 | 0 | 0 | 0 | 0 | 0 | 0 | 0.05 | 0.05 | 0.01 | 0.04 | 0 | 0 | 0 | 0 | 0 | 0.01 |
| Thysanoptera | 0 | 0 | 0 | 0 | 0 | 0 | 0 | 0 | 0 | 0 | 0 | 0 | 0 | 0 | 0 | 0 | 0 | 0.05 | 0.05 | 0.01 | 0.04 | 0 | 0 | 0 | 0 | 0 | 0.01 |
| Diptera.larvae | 0 | 0 | 0 | 0 | 0 | 0 | 0 | 0 | 0 | 0 | 0 | 0 | 0 | 0 | 0 | 0 | 0 | 0.05 | 0.05 | 0.01 | 0.04 | 0 | 0 | 0 | 0 | 0 | 0.01 |

**Table S2**. The attributes of taxa that co-determine the relative strengths in the interaction matrix. “above”, “epi”, “hemi”, “eu” refer to the vertical stratification of a group. All attributes except body mass were taken from Potapov (2022)

| taxon | MeanMass.mg | StDMass.mg | Agility | PhysicalProtection | Metabolites | above | epi | hemi | eu |
| --- | --- | --- | --- | --- | --- | --- | --- | --- | --- |
| Bacterivore.nematodes | 0.000487 | 0.00317 | 1 | 1 | 1 | 0 | 0 | 1 | 1 |
| Fungivore.nematodes | 0.000383 | 0.000438 | 1 | 1 | 1 | 0 | 0 | 1 | 1 |
| Herbivore.nematodes | 0.000552 | 0.022337 | 1 | 1 | 1 | 0 | 0 | 1 | 1 |
| Omnivore.nematodes | 0.002135 | 0.016611 | 1 | 1 | 1 | 0 | 0 | 1 | 1 |
| Predator.nematodes | 0.00349 | 0.004634 | 1 | 1 | 1 | 0 | 0 | 1 | 1 |
| Protura | 0.012832 | 0.006904 | 1 | 1 | 1 | 0 | 0 | 1 | 1 |
| Pauropoda | 0.010055 | 0.006513 | 1 | 1 | 1 | 0 | 0 | 1 | 1 |
| Symphyla | 0.115804 | 0.091635 | 1 | 1 | 0.4 | 0 | 0 | 1 | 1 |
| Oribatida | 0.012428 | 0.012256 | 1 | 0.4 | 0.7 | 0 | 0 | 1 | 0.5 |
| Mesostigmata | 0.00559 | 0.008244 | 1 | 0.7 | 1 | 0 | 0 | 1 | 0.5 |
| Prostigmata | 0.002877 | 0.004196 | 1 | 0.7 | 1 | 0 | 0.5 | 1 | 0.5 |
| Edaphic.Entomobryomorpha | 0.018402 | 0.026293 | 0.7 | 1 | 1 | 0 | 0 | 1 | 0.5 |
| Edaphic.Neelipleona | 0.001299 | 0.000682 | 0.7 | 1 | 1 | 0 | 0 | 1 | 0.5 |
| Edaphic.Poduromorpha | 0.008425 | 0.00718 | 0.7 | 1 | 1 | 0 | 0 | 1 | 0.5 |
| Epigeic.Symphypleona | 0.002524 | 0.002563 | 0.7 | 1 | 1 | 0.5 | 1 | 0.5 | 0 |
| Epigeic.Entomobryomorpha | 0.032618 | 0.019112 | 0.7 | 1 | 1 | 0.5 | 1 | 0.5 | 0 |
| Epigeic.Poduromorpha | 0.015901 | 0.014885 | 0.7 | 1 | 1 | 0.5 | 1 | 0.5 | 0 |
| Thysanoptera | 0.13635 | 0.165533 | 1 | 1 | 1 | 0 | 1 | 0 | 0 |
| Chilopoda | 4.337995 | 7.530042 | 1 | 1 | 1 | 0 | 0.5 | 1 | 0.5 |
| Diplopoda | 10.43434 | 15.47354 | 1 | 0.4 | 0.7 | 0 | 1 | 1 | 0.5 |
| Isopoda | 10.4347 | 4.06558 | 1 | 0.4 | 1 | 0 | 1 | 0.5 | 0 |
| Araneae | 2.672747 | 8.858881 | 1 | 1 | 1 | 0.5 | 1 | 0.5 | 0 |
| Staphylinidae | 1.051027 | 1.956718 | 1 | 0.4 | 0.4 | 1 | 1 | 1 | 1 |
| Hemiptera | 0.668405 | 1.566864 | 0.7 | 0.7 | 0.7 | 0.5 | 1 | 0.5 | 0.5 |
| Gastropoda | 29.07547 | 51.57957 | 1 | 0.4 | 0.4 | 0.5 | 1 | 1 | 0.5 |
| Coleoptera | 1.716337 | 2.958803 | 1 | 0.4 | 0.4 | 1 | 1 | 1 | 1 |
| Diptera.larvae | 0.249 | 0.0578 | 1 | 1 | 1 | 1 | 1 | 1 | 0 |

The use of body-mass information is described in Figure S1. The vectors Agility, PhysicalProtection and Metabolites down-weight the probability of an interaction with a given prey, in the presence of the relevant attribute. The vertical stratification vectors were used to construct a Bray-Curtis dissimilarity matrix, which reflects the probability of encounter between taxa based on their vertical stratification.


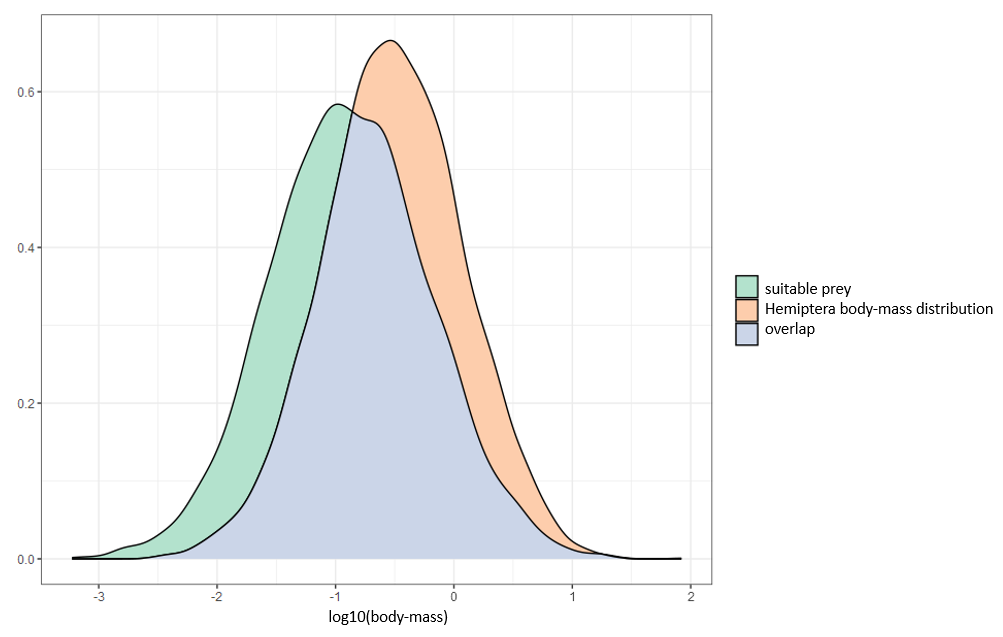


**Figure S1** Schematic representation of the calculation of the probability of a predator consuming certain prey taxa based on body-mass, using Araneae and Hemiptera as an example. Suitable prey body-mass distribution was derived from the predator body-mass distribution assuming PPMR = 3.98 (10^.6). The overlap of the body-mass distribution of a potential prey taxon with the suitable prey distribution determines the probability of consuming that prey taxon.

**Incorporation of diet uncertainty using the Dirichlet distribution**

The Dirichlet distribution is a multivariate probability distribution used for proportions of two or more component categories (in our case types of resource or prey taxa). It is parameterized by a vector of positive real numbers which express the component probabilities. The components are constrained to sum to one. A vector (10, 30, 60) expresses the expectation that the first component is 10% of the whole, the second 30% and the third 60%. A vector (100, 300, 600) expresses the same expectation but with higher concentration (i.e. reduced uncertainty) around the expected proportions. Figure S2 shows three examples of the same expected proportions with increasing uncertainty


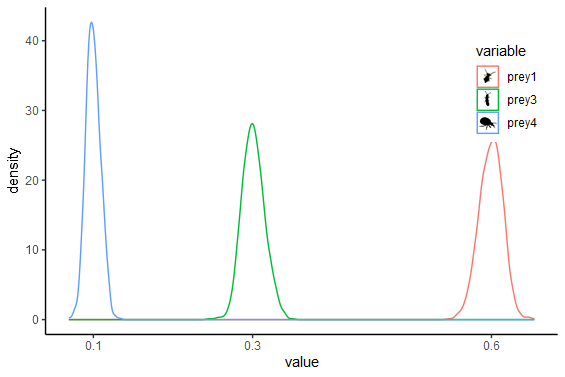


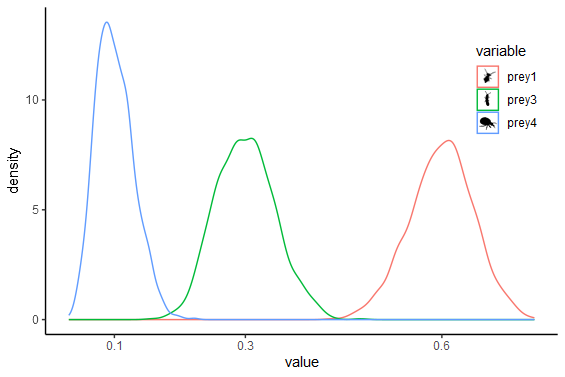


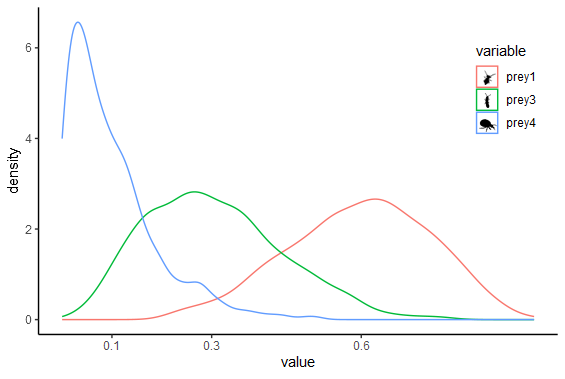


**Figure S2** Example of low (top), intermediate (middle) and high (bottom) consumer diet uncertainty, for a hypothetical consumer with an expected diet composition of (0.1, 0.3, 0.6) of three prey taxa.


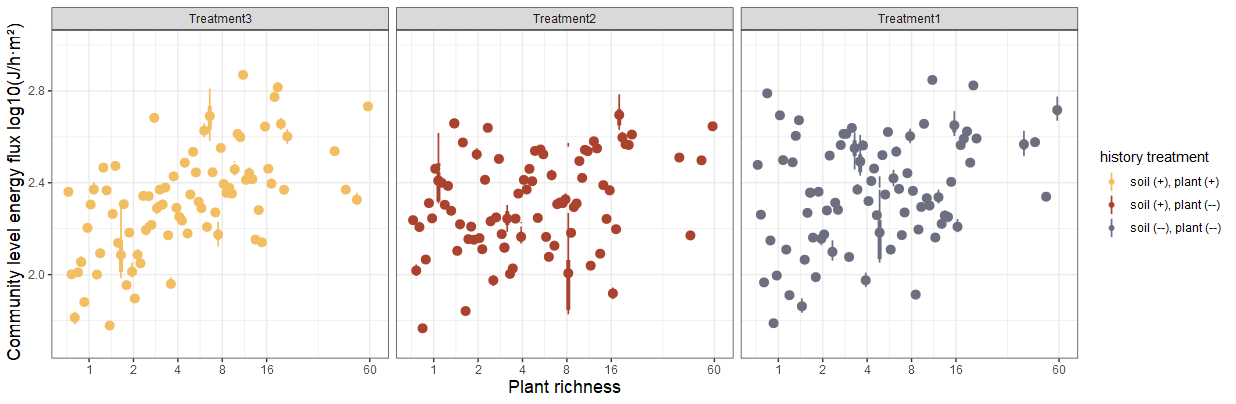


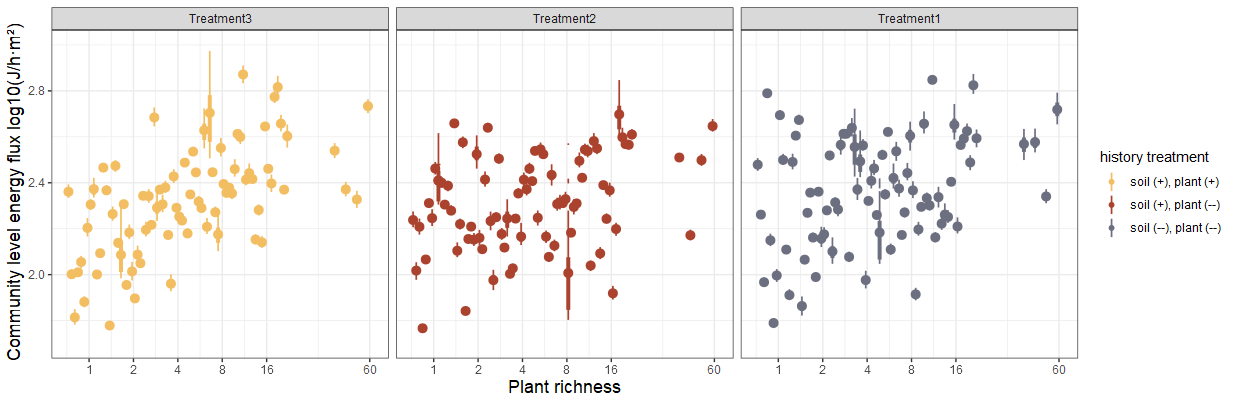


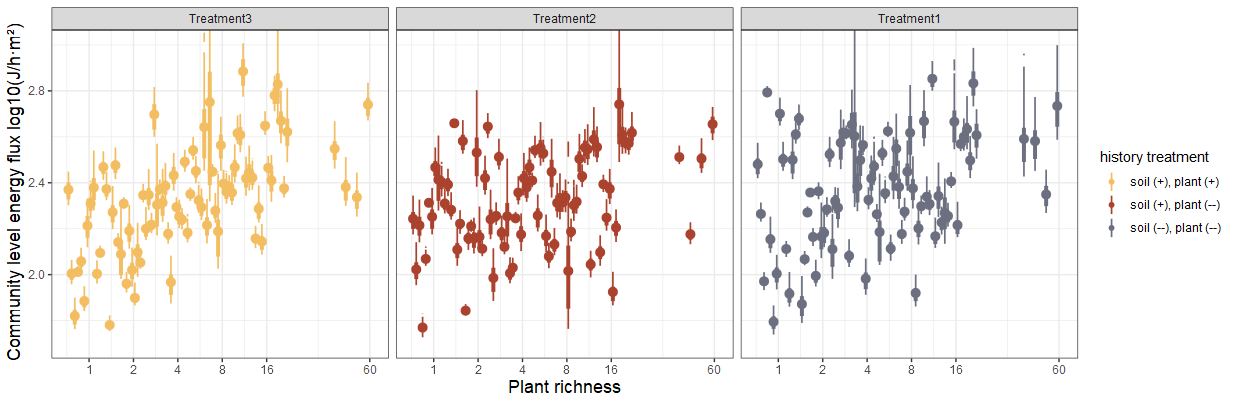


**Figure S3** The effect of low (top), intermediate (middle) and high (bottom) consumer diet uncertainty on community level energy flux. The color scheme is the same as in the main figures. For a given level of diet uncertainty, some food-webs are more sensitive (variable) than others.

Food-webs exhibited varying sensitivity to diet uncertainty, in terms of the resulting flux uncertainty (Figure S3). However, incorporating diet uncertainty had negligible effects on model estimates (Figure S4). This indicates that the extent of flux uncertainty is not associated with variables of interest, but rather is randomly distributed in food-webs across the range of the dependent and the independent variables.



**Figure S4** Model coefficients excluding diet uncertainty (-) and at low (1000), intermediate (100) and high (10) uncertainty. Each column corresponds to a model. Despite the effects of diet uncertainty on energy fluxes as shown in Figure S3, model coefficients remained practically unchanged. Points are mean estimates bound by 90% credible intervals.

**
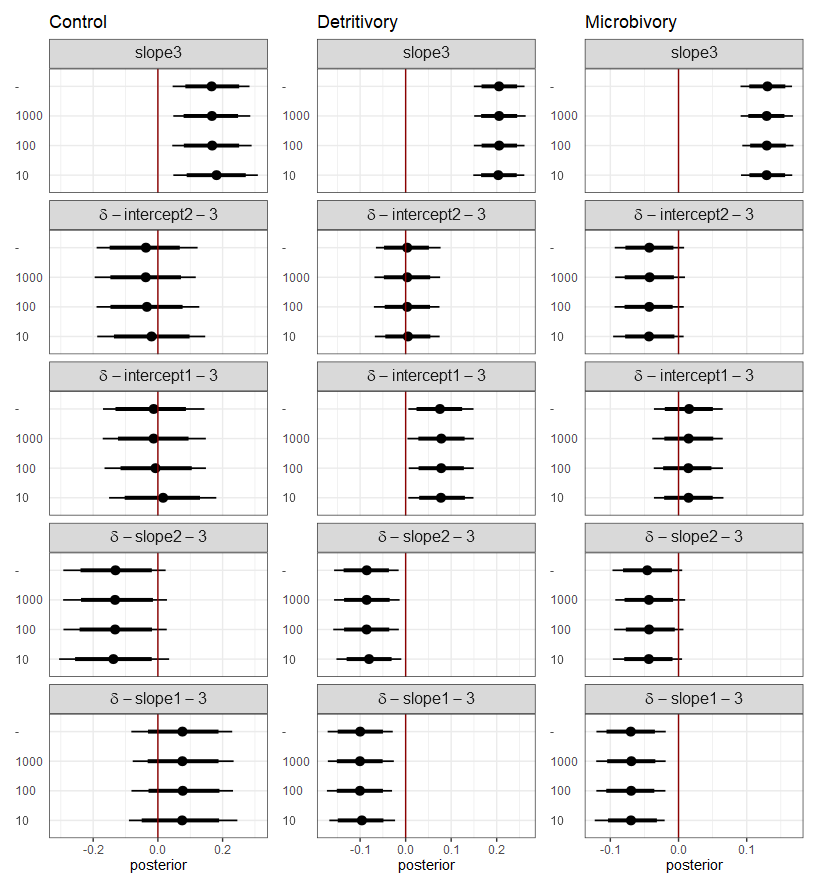
**

**Figure S4** (continued)


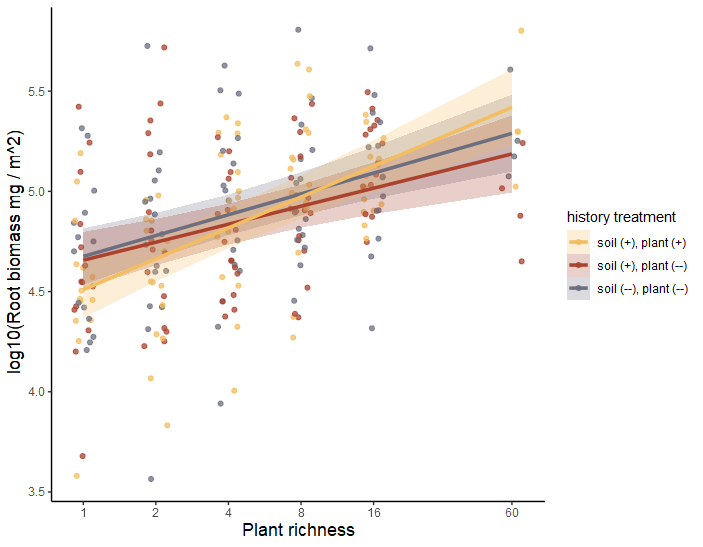


**Figure S5** The relationship between plant richness and root biomass in the 0-5 cm depth soil layer. Lines show mean estimates for the average relationship bound by 90% uncertainty intervals.


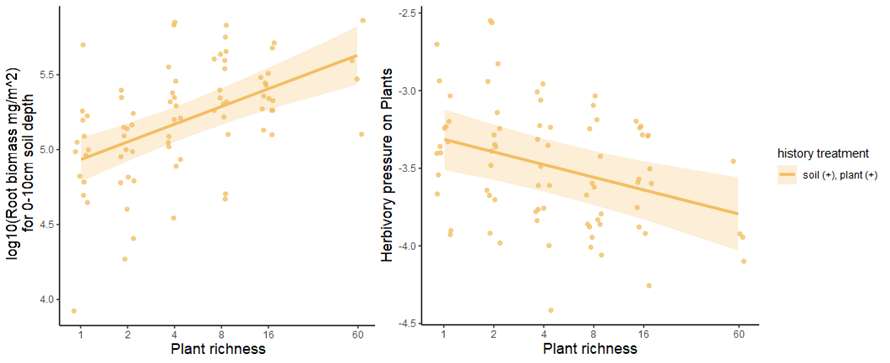


**Figure S6** The relationship of plant richness and root biomass in the 0-10 cm depth soil layer in control subplots (left). The relationship between plant richness and herbivory pressure on plants, using the full 0-10 cm depth root biomass (right). Lines show mean estimates for the average relationship bound by 90% uncertainty intervals.

Root biomass in the 0-10 cm depth increased with plant richness in the control plots (Fig. S6, left). The relationship of plant richness and herbivory pressure in control plots is shallower when we incorporate the 0-10 cm root biomass but remains statistically clear (Fig. S6, right; mean slope [90% HPD] = -0.13 [-0.20, -0.06] compared to -0.19 [-0.265, -0.11]).

The mismatch of sampling depth for soil fauna (0-10 cm) and root biomass (0-5 cm) means that, in our main analysis, we are overestimating herbivory pressure in absolute terms. Additionally, communities with plant history may have more root biomass at lower depths compared to communities without plant history. Therefore this overestimation may be more pronounced in our control communities, which in turn might have obscured differences between control and history treatments.

**Tables S3 – S4** Information on the creators and license of the silhouettes used in Figure 1 of the main text

| **Taxon** | **In Phylopic.org** | **Creator** | **License** |
| --- | --- | --- | --- |
| Collembola | Sminthuridae | Birgit Lang | Attribution 3.0 Unported |
| Collembola | Tetrodontophora bielanensis | Birgit Lang | Attribution 3.0 Unported |
| Collembola | Mesaphorura | Birgit Lang | Attribution 3.0 Unported |
| Gamasida | Multidentorhodacarus squamosus | Birgit Lang | Attribution 3.0 Unported |
| Protura | Protura | Birgit Lang | Attribution 3.0 Unported |
| Chilopoda | Lithobius forficatus | Birgit Lang | CC0 1.0 Universal Public Domain Dedication |
| Coleoptera | Ocypus | Birgit Lang | CC0 1.0 Universal Public Domain Dedication |
| Nematodes | Oscheius dolichura | Birgit Lang | CC0 1.0 Universal Public Domain Dedication |
| Prostigmata | Tetranychus urticae | Christoph Schomburg | CC0 1.0 Universal Public Domain Dedication |
| Isopoda | Armadillidium vulgare | Christoph Schomburg | CC0 1.0 Universal Public Domain Dedication |
| Hemiptera | Aphididae | Christoph Schomburg | CC0 1.0 Universal Public Domain Dedication |
| Syphyla | Scutigerella immaculata | Gemma Martínez-Redondo | CC0 1.0 Universal Public Domain Dedication |
| Diplopoda | Julida | Gemma Martínez-Redondo | CC0 1.0 Universal Public Domain Dedication |
| Pauropoda | Pauropus huxleyi | Gemma Martínez-Redondo | CC0 1.0 Universal Public Domain Dedication |
| Oribatida | Hypochthonius rufulus | Kamil S. Jaron | CC0 1.0 Universal Public Domain Dedication |
| Nematodes | Pratylenchus | Kamil S. Jaron | CC0 1.0 Universal Public Domain Dedication |
| Collembola | Orchesella cincta | Kamil S. Jaron | CC0 1.0 Universal Public Domain Dedication |
| Collembola | Folsomia candida | Kamil S. Jaron | CC0 1.0 Universal Public Domain Dedication |
| Spiders | Atrax robustus | Margot Michaud | CC0 1.0 Universal Public Domain Dedication |
| Diptera | Tipula | Michael Day | CC0 1.0 Universal Public Domain Dedication |
| Fungi | Rhizopus oryzae | T. Michael Keesey | CC0 1.0 Universal Public Domain Dedication |
| Coleoptera | Agriotes mancus | T. Michael Keesey | Public Domain Mark 1.0 |
| Mollusca | Helix aspersa | Yan Wong | CC0 1.0 Universal Public Domain Dedication |

|  | **Source** | **License** |
| --- | --- | --- |
| roots | https://svgsilh.com/image/311540.html | CC0 1.0 Universal Public Domain Dedication |
| detritus | https://svgsilh.com/image/576619.html | CC0 1.0 Universal Public Domain Dedication |
| bacteria | https://svgsilh.com/image/296502.html | CC0 1.0 Universal Public Domain Dedication |
